# Iron Metabolism Disorders for Cognitive Dysfunction After Mild Traumatic Brain Injury

**DOI:** 10.3389/fnins.2021.587197

**Published:** 2021-03-16

**Authors:** Suna Huang, Su Li, Hua Feng, Yujie Chen

**Affiliations:** ^1^Department of Neurosurgery, Southwest Hospital, Third Military Medical University (Army Military Medical University), Chongqing, China; ^2^State Key Laboratory of Trauma, Burn and Combined Injury, Southwest Hospital, Third Military Medical University (Army Military Medical University), Chongqing, China; ^3^Chongqing Key Laboratory of Precision Neuromedicine and Neuroregenaration, Third Military Medical University (Army Military Medical University), Chongqing, China

**Keywords:** traumatic brain injury, cognitive dysfunction, cerebrospinal-fluid contacting neuron, autophagy, iron metabolism

## Abstract

Traumatic brain injury (TBI) is one of the most harmful forms of acute brain injury and predicted to be one of the three major neurological diseases that cause neurological disabilities by 2030. A series of secondary injury cascades often cause cognitive dysfunction of TBI patients leading to poor prognosis. However, there are still no effective intervention measures, which drive us to explore new therapeutic targets. In this process, the most part of mild traumatic brain injury (mTBI) is ignored because its initial symptoms seemed not serious. Unfortunately, the ignored mTBI accounts for 80% of the total TBI, and a large part of the patients have long-term cognitive dysfunction. Iron deposition has been observed in mTBI patients and accompanies the whole pathological process. Iron accumulation may affect long-term cognitive dysfunction from three pathways: local injury, iron deposition induces tau phosphorylation, the formation of neurofibrillary tangles; neural cells death; and neural network damage, iron deposition leads to axonal injury by utilizing the iron sensibility of oligodendrocytes. Thus, iron overload and metabolism dysfunction was thought to play a pivotal role in mTBI pathophysiology. Cerebrospinal fluid-contacting neurons (CSF-cNs) located in the ependyma have bidirectional communication function between cerebral–spinal fluid and brain parenchyma, and may participate in the pathway of iron-induced cognitive dysfunction through projected nerve fibers and transmitted factor, such as 5-hydroxytryptamine, etc. The present review provides an overview of the metabolism and function of iron in mTBI, and to seek a potential new treatment target for mTBI with a novel perspective through combined iron and CSF-cNs.

## Introduction

Traumatic brain injury (TBI) is a general term for pathological or structural changes in the brain caused by impact, bumps, penetrating injuries, or blasting under external forces (Menon et al., 2010; Cook and Hawley, 2014; Pavlovic et al., 2019), which involves at least one of the following symptoms: loss or a decreased level of consciousness, loss of memory for events before or after the injury, neurological deficits (weakness, loss of balance, changes in vision, etc.), or alterations in mental state (confusion, disorientation, slowed thinking, etc.). Based on the clinical awareness level of the Glasgow Coma Scale (GCS), the severity of TBI is usually classified as mild (mTBI) (13–15 points), moderate (9–12 points), or severe (3–8 points). The primary and secondary injuries of trauma may cause severe and long-lasting damage to TBI patients.

In the acute phase, moderate and severe TBI often cause intracranial hemorrhage, and half of the TBI patients may suffer from traumatic cerebral hemorrhage in addition to the primitive hematoma that causes compressive damage to brain tissue (Kurland et al., 2012). Some acute clinical symptoms, like sharply increased intracranial pressure and cerebral hernia, lead to the mortality of sever TBI as much as 30–40% (Rosenfeld et al., 2012). Due to the severe clinical symptoms of this part of TBI patients, neurosurgeons always administrate posttraumatic intracranial hematoma (TICH) removal to prevent increased intracranial pressure and cerebral hernia (Tao et al., 2017). Basing on the secondary injury mechanism, a proportion of severe TBI survivors have long-term physical and cognitive disorder, but after high quality care in the emergency or ICU, the consequence may be attenuated.

Reversely, mTBI, accounting for more than 80% of all TBI patients, is often ignored, as it is generally non-fatal (0.1% lethality) (Af Geijerstam and Britton, 2003). MTBI mainly consists of sport-related TBI and military-related TBI. Sport-related mTBI has the highest incidence (approximately 20%) among all types of TBI (Theadom et al., 2014). Except the professional athletes, due to the benefits for physical and mental health, more and more people participating in sports, and the incidence of global sports-related TBI is continuously rising (Daneshvar et al., 2011). From 2007 to 2011, approximately 84% of military-related TBIs were classified as mTBIs (Mondello et al., 2014); military personnel exposed to explosive device blasts on the battlefield are especially prone to TBI, and this incidence is closely related to international military situations. Many single-event mTBI patients experience symptoms or disability even for more than 1 year (Nelson et al., 2019), including chronic dizziness, fatigue, and headache (Silver et al., 2009), which are collectively referred to as postconcussive syndrome (Sterr et al., 2006; Dikmen et al., 2010).

Many professional athletes or veterans have a history of repetitive head impacts and are likely to develop chronic traumatic encephalopathy (CTE) due to multiple episodes of mTBI (McKee et al., 2015; Stein et al., 2015). Compared with single-event mTBI, repeat mTBI will induce more serious neurological dysfunction (Ayubcha et al., 2021). With passage of time, CTE patients gradually present with cognitive impairment and memory loss, and at last, dementia is virtually inevitable (McKee et al., 2013). Cognitive dysfunction is often observed after TBI and is the major complication of TBI during the subacute or chronic phase. Cognitive dysfunction in patients with mTBI can usually last for 3–6 months or longer (Whiteneck et al., 2004; Boake et al., 2005; Roe et al., 2009). Due to the inconsistency of research methods and the different subtypes of patients included, conflicting results on cognitive dysfunction may be reported. However, it was consistently reported in all studies that the impact of post-TBI cognitive dysfunction on patients’ work, daily life, and social functions as well as the burden on families and society cannot be ignored (Gustavsson et al., 2011). To date, the mechanism of early cognitive dysfunction after TBI is still unclear (Wolf and Koch, 2016; Zhao et al., 2017).

Chronic traumatic encephalopathy is a condition that significantly shortens the life span (Wortzel et al., 2013). Autopsy revealed that the most typical pathological feature of CTE is excessive deposition of tau protein and formation of neurofibrillary tangles (McKee et al., 2015; Kanaan et al., 2016). Excessive iron deposits were observed in these nerve fiber tangles, where free iron can induce tau phosphorylation through oxidative stress (Bouras et al., 1997; Hui et al., 2011). Coincidently, earlier reports revealed the deposition of non-heme iron in the deep cortex and hippocampus in the postmortem brain tissues of mTBI patients (Bouras et al., 1997). Thus, iron gradually becomes the intervention target in TBI.

## Current Understanding of Secondary Brain Injury After Traumatic Brain Injury

**FIGURE 1:**
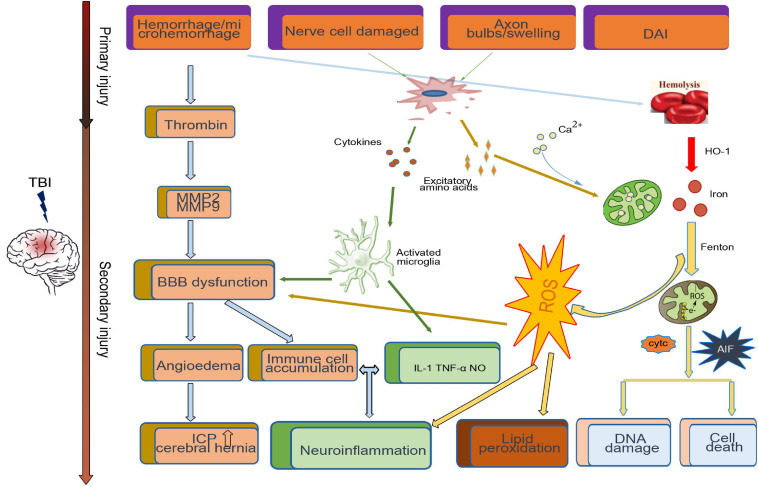
Primary and secondary pathophysiology in traumatic brain injury. Immediately at the time of impact, the brain suffers a direct mechanical trauma, which induced TBI primary injury including hemorrhage/microhemorrhage, cell death, diffuse axonal injury, etc. Hours to days after impact, the secondary injury initiated by the primary injury, such as the microglia activated by cytokines and excitatory amino acids from damaged cell, cooperates with the dysfunctional blood–brain barrier (BBB)-induced immune cell accumulation and neuroinflammation. Excessive iron and calcium-mediated mitochondrial dysfunction increase the generation of free radical and oxidative species, which induce lipid peroxidation, DNA damage, and cell death.

After TBI, the primary injury leads to neuronal death around the injury site. A series of secondary injury cascades, such as the release of blood metabolites, microglial activation, thrombin activity, the release of excitatory amino acids, and proinflammatory factors, are initiated (Figure 1). Patients with various degrees of TBI may suffer from a long period of secondary injury, which plays a key role in determining the final severity and recovery of nerve injury after TBI (Lee et al., 2015).

### Inflammation

Neuroinflammation is a key pathophysiological mechanism of secondary injury, as it can produce neurotoxic effects on neurons through oxidative stress, apoptosis, and excitotoxicity (Faden et al., 2016). After TBI, there is an activation of neuroinflammation response pathway mediated by microglia cells that were not initially damaged, and the activated microglial cells may promote the release of proinflammatory factors such as interleukin 1, tumor necrosis factor, nitric oxide, etc., (Kumar and Loane, 2012), inducing a progressive cycle of chronic inflammation (Borlongan et al., 2015). Except microglia activation, the ROS generated after TBI and peripheral immune cells like macrophages, neutrophils, and lymphocytes recruit to the injured site through the disrupted blood–brain barrier (BBB), which are key components to exacerbate the inflammation (Sulhan et al., 2020).

### Blood–Brain Barrier Destroyed

The BBB is crucial in maintaining homeostasis within the brain’s biochemical environment. After TBI, activation of microglia and the cytokines released by damaged cells destroy tight junctions through impairment of BBB endothelial cells, pericytes, astrocytes, and through the synthesis of tight junction proteins; this leads to an increase in BBB permeability, subsequent edema, and the accumulation of plasma-derived factors and immune cells in brain tissue (Shetty et al., 2014; Sulhan et al., 2020), leading to a sharp increase in intracranial pressure and eventually cerebral hernia (Winkler et al., 2016).

### Excitatory Amino

Similarly, increased release of excitatory amino acids is also involved in nerve function damage after TBI. Increased release of glutamate from presynaptic terminals activates *N*-methyl-D-aspartate (NMDA) receptors on postsynaptic terminals to lead to a widespread neuronal depolarization or spreading depression, further disrupting the sodium ion, potassium ion, and calcium ion equilibria (Bouley et al., 2019). Then, the function of the mitochondria, which is the calium regulator, is hampered by abnormally elevated intracellular calium (Langlois et al., 2006). As a result of such abnormalities, the membrane pump is continuously activated to correct the example level of imbalance, consuming a large amount of glucose and generating a large amount of lactic acid, leading to acidosis and edema (Zetterberg et al., 2013; Barkhoudarian et al., 2016).

Basing on the secondary injury mechanism, anti-oxidation and anti-excitatory amino have been used in releasing the neurological dysfunction, not showing effect, and currently, there are no effective treatments approved by clinical trials for TBI patients (Pearn et al., 2017).

### Iron

Imaging examination found a subgroup of mTBI patients with microbleeding (Nisenbaum et al., 2014). Long time, continuous, or discontinuous microbleeding can induce red blood cells, and thrombin enters the brain continuously. Iron is released into the brain tissue 24 h after the onset of bleeding (Xiong et al., 2013); on the seventh day, the non-heme iron content is tripled and can remain at high levels for 28 days in the brain (Xiong et al., 2013). The evidence suggested that increased iron accumulation was negatively associated with cognitive outcomes in chronic TBI patients (Lu et al., 2015). With the formation of a membrane attack complex, the complementary cascade reaction may be a mechanism of thrombin-induced brain damage, resulting in the lysis of red blood cells (Gong et al., 2005). Catalyzed by HO-1, heme-bound iron is released as free iron and contributes to the nerve injury process (Beschorner et al., 2000). Therefore, due to iron deposition accompany with the whole pathological process, iron is considered an important pathophysiological factor involved in these secondary injuries after mTBI (Nisenbaum et al., 2014).

## Iron Metabolism

### Normal Brain Iron Metabolism

Iron is necessary for brain metabolism and is the most abundant metal in the brain. Iron plays an essential role in myelination, oxygen transport, neurotransmitter transmission, and mitochondrial energy production (Salvador, 2010; Ward et al., 2014).

The BBB plays a vital role in brain iron uptake and protects the brain from fluctuations in iron levels throughout the body. Iron in the circulatory system enters the brain through the BBB mainly in two forms: a transferrin-bound iron-transferrin receptor complex and a non-transferrin-bound iron (Singh et al., 2014). Studies have reported that choroid plexus endothelial cells also regulate the entry of iron into the central nervous system (Rouault and Cooperman, 2006).

Astrocytes redistribute iron, which have gotten through the BBB to other cells in the central nervous system with the assistance of ceruloplasmin (Dringen et al., 2007). Ceruloplasmin can oxidize ferrous iron to Fe^3+^, which binds to transferrin in the cerebral spinal fluid (CSF) (Singh et al., 2014). In addition, there is a large amount of non-transferrin-bound iron circulating in the CSF together with ATP, citrate, and ascorbic acid in cells that do not express transferrin receptors (Malecki et al., 1999; Ke and Qian, 2007). Oligodendrocytes have the highest iron content in the brain (Connor and Menzies, 1996), which may explain their high sensitivity to oxidative stress (Smith et al., 1999). Although oligodendrocytes are mainly responsible for transferrin expression and secretion, they take non-transferrin-bound iron in the form of ferritin via the Tim-2 receptor; astrocytes take non-transferrin-bound iron via DMT1 (Rouault and Cooperman, 2006; Todorich et al., 2008; Lane et al., 2010). Neurons take iron from the CSF and interstitial fluid by transferrin-bound iron.

Part of the cytoplasmic iron is involved in the normal metabolic activities of the cell, such as the activity of mitoferrin expressed in the mitochondrial inner membrane, which transports iron from the cytoplasm to the mitochondria to participate in the synthesis of heme and iron–sulfur clusters (Benarroch, 2009). Some iron in the form of a complex is directly transferred to ferritin through the PCBP1 protein–protein effect (Philpott et al., 2017). A large portion of cytoplasmic iron is contained in the lysosome (Yu et al., 2003). Apart from the iron serving as co-factors and the iron stored in ferritin, the remaining divalent iron in the cytoplasm (a concentration of approximately 0.5–5 μm) is called the free iron pool (Espósito et al., 2002; Ma et al., 2006; Bayır et al., 2020). Under normal circumstances, >95% of the ferrous iron in the cytoplasm is bound with GSH at a ratio of 1:1 and forms a stable iron complex to prevent free Fe^2+^ from generating ROS by the Fenton reaction to damage cells (Patel et al., 2019).

The uptake and storage of cellular iron mainly depend on the regulatory effects of the cytoplasmic protein iron regulatory protein (IRP)1 and IRP2 (Ponka, 1999; Styś et al., 2011). When cells experience iron deficiency, IRPs bind to the iron-responsive element (IRE) of ferritin mRNA and inhibit the translation of ferritin to reduce iron storage. Under the mediation of nuclear receptor coactivator 4 (NCOA4), ferritin is transported to the lysosome and degraded through selective autophagy to release free iron for cell utilization (Quiles del Rey and Mancias, 2019). On the other hand, IRPs combine with the IRE on the transferrin receptor mRNA to stabilize its mRNA, promote the expression of transferrin receptor, and increase iron uptake (Recalcati et al., 2010; Wagner et al., 2016). Meanwhile, excessive cellular iron may trigger the opposite regulation mechanism. Similar to peripheral tissues, brain tissues also transport iron out of cells under the synergistic effect of Fpn/hephaestin and/or Fpn/CP (Li and Qian, 2002; Rouault and Cooperman, 2006). Brain iron is reabsorbed from the subarachnoid space back into the blood through the CSF.

### Iron Metabolism Disorder Under Traumatic Brain Injury Condition

**FIGURE 2:**
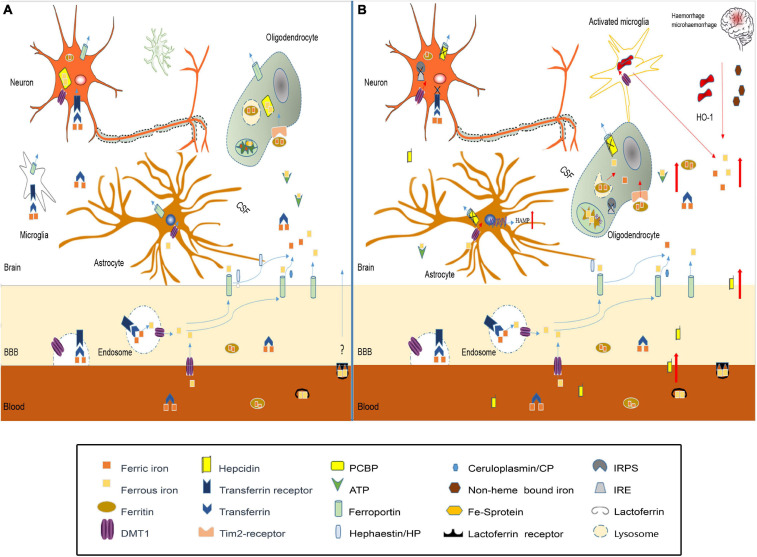
Iron metabolism disorder in traumatic brain injury. **(A)** In normal brain, iron transport across the luminal membrane of the BBB mainly relies on the transferrin/transferrin receptor (Tf/TfR) pathway, Tf-TfR-bounding iron complex under the help of divalent metal transporter 1 (DMT1) released from the endosome then transported across the abluminal membrane by ferroportin 1/hephaestin (Fpn1/Heph) and/or Fpn1/ceruloplasmin (CP). Non-transferrin-bound iron can be transported across the BBB by DMT1, etc. After release from the microvascular endothelial cells through Fpn1, iron mainly in the forms of Tf-fe^3+^, ferritin-Fe^3+^, and ATP-Fe^2+^, circulates in the cerebral spinal fluid (CSF), which is very convenient to be utilized by nerve cells. Astrocytes take iron via DMT1, and oligodendrocytes take iron in the form of ferritin via the Tim-2 receptor. Neurons and microglias expressing TfR can take iron from the CSF through TfR and DMT1. Cytoplasm iron: (1) Participates in cell metabolic activities, such as the synthesis of heme in mitochondria. (2) Contained in poly(rC)-binding protein (PCBP), ferritin, lysosome to prevent free iron from generating ROS. **(B)** In traumatic brain injury, iron accumulation occurs in the situation of hemorrhage/microhemorrhage. Excessive iron suppresses iron regulatory proteins (IRPS) combined with TfR and Fpn1, ferritin iron regulatory elements (IRES) induce TfR decrease and Fpn1, and ferritin increased to prevent neuron iron overload, but brain hepcidin expression is increased including local and peripheral hepcidin, which is transported into the brain through the dysfunctional BBB. Hepcidin internalizes Fpn1 to suppress the output of iron. At the same time, increased DMT1 enhances iron uptake in all nerve cells except oligodenrocytes, but increased ferritin promotes oligodenrocytes and takes iron through the Tim2 receptor. A large amount of increased cytoplasm iron is transported into the mitochondria generating ROS-induced organelle damage including lysosome and cell death.

Studies have confirmed that iron homeostasis in the brain is disrupted after TBI (Figure 2). The possible causes of brain iron metabolism disorder may include iron accumulation caused by red blood cell lysis, iron metabolism-related protein changes, mitochondria, and lysosome dysfunction.

Iron accumulation is observed after TBI (Raz et al., 2011; Liu et al., 2013), which is consistent with the fact that moderate to severe TBI and many cases of mTBI have cerebral hemorrhage. Under the effect of HO-1, iron is released from heme as free iron. Microglia, as brain-specific phagocytic cells, will engulf red blood cells, and then release degraded iron into the interstitium of the brain (Andersen et al., 2013); these cells are all a source of excessive iron in the brain after TBI. The increased iron in the interstitium and CSF will be transported to various types of nerve cells in various forms, eventually leading to iron accumulation in nerve cells, which can produce large amounts of ROS to intensify oxidative stress and aggravate secondary damage after TBI (Nunez et al., 2012).

The possible causes of brain iron accumulation may also include the expression of iron metabolism-related proteins changes. Studies have shown that the expression of ferritin, transferrin, and transferrin receptors in the brain is increased after intracranial hemorrhage (Wu et al., 2003), which may increase iron absorption in neurons and oligodendrocytes, which is consistent with previous reports that iron accumulates in brain cells after TBI. In the case of inflammation, McCarthy et al. (2018) found that an acidic environment increased the expression of DMT1 in microglia and increased the uptake of non-transferrin-conjugated iron. Additionally, excessive iron can inhibit IRPs in cells, then regulating the increased expression of ferritin and Fpn1 to enhance the storage capacity and iron output capacity for the increased iron pool (Thirupathi and Chang, 2019; Wang et al., 2019). However, when inflammation and iron overload in cells occur, the expression of hepcidin is also increased (Lieblein-Boff et al., 2013; Rochette et al., 2015). Studies have shown that hepcidin can enter the central nervous system through the BBB (Raha-Chowdhury et al., 2015), which, with increased permeability after TBI, may allow a large amount of hepcidin to the brain tissue; thus, hepcidin internalizes Fpn1, which affects the iron output. As a result, iron uptake capacity is enhanced, while iron output capacity is weakened, aggravating the accumulation of iron in cells.

In addition, the possible causes of brain iron metabolism disorder may include cell mitochondrial and lysosomal dysfunction. Under pathological conditions of TBI, the MCU protein on the inner mitochondrial membrane can transport a large amount of calcium ions into the mitochondria, disrupting the normal function of the mitochondria (Peng and Jou, 2010; Zhang et al., 2019). When MCUs rapidly mediate calcium ions into the mitochondria, they also simultaneously mediate a large amount of iron into the mitochondrial matrix (Zhang et al., 2019), which can cause iron homeostasis disorder. Excessive iron generates ROS through the Fenton reaction, which further aggravates mitochondrial dysfunction and forms a progressive vicious cycle. Using MCU inhibitors can promote mitochondrial dysfunction recovery caused by iron overload by reducing ROS production and mitochondrial depolarization (Sripetchwandee et al., 2013). In normal cells, a large portion of iron is contained in lysosomes in the form of divalent iron (Yu et al., 2003). After TBI, oxidative stress in cells can cause damage to certain lysosomes. The sensitivity to oxidative stress is related to the amount of iron contained in lysosomes (Terman and Kurz, 2013). The damaged lysosome ruptures and releases large amounts of iron into the cytoplasm.

## Iron-Related Brain Injury After Traumatic Brain Injury

### Local Injury

Iron deposition in the brain could induce local pathological appearance. Previous studies have observed iron deposition in CTE patients. The main pathological feature of CTE is the abnormal aggregation of tau protein in neurons, astrocytes, and cell synapses in the deep cortical sulci. In this process, accumulated iron promotes the phosphorylation of tau protein in the brains of CTE patients through oxidative stress and aggregates to form nerve fiber tangles (Shen, 2015). Studies have shown that the sustained occurrence of mild TBI can increase the risk of Parkinson’s disease in patients over 55 years old in 5–7 years (Gardner et al., 2015), which may also be related to excessive iron deposition after TBI.

### Neural Network Injury

There is a hypothesis that damage to the brain network, mainly axons, may be the basis of cognitive dysfunction after TBI (Wolf and Koch, 2016), and iron may play an important role in the process. Despite the primary injury due to mechanical force, diffuse and multifocal damage often appears, leading to diffuse axonal injury by blunt force of shearing, tearing, and stretching of the axon and further damage of the long-distance white matter connection, with large-scale network disconnection as the core mechanism of cognitive impairment. Diffuse axonal injury induced by initial mechanical force could lead to cognitive decline immediately when the TBI occurs and is always irreversible. Besides the initial mechanical force, the complex secondary cascade always induces long-term diffuse axonal injury. This characteristic is consistent with TBI especially that mTBI patients have long-term cognitive dysfunction (Mu et al., 2019).

The mixed and intertwined axon and myelin is one pathology nature in diffuse axonal injury. Oligodendrocytes provide an important biological basis for neural network integration and high-level functions. Oligodendrocyte loss following TBI takes place. Demyelinated axons are prone to damage, so the loss of oligodendrocytes may lead to secondary axonal injury (Armstrong et al., 2016). Oligodendrocytes have the highest iron content in the brain and are highly sensitive to oxidative stress and iron accumulation. A recent study demonstrated that iron homeostasis disorder and ferroptosis can induce oligodendrocyte loss and demyelination, and iron chelation can rescue iron-mediated oligodendrocyte death (Nobuta et al., 2019). Thus, iron accumulation and metabolism disorder after TBI may lead to secondary axonal injury and cognitive dysfunction through inducing oligodendrocyte apoptosis.

### Cells Death

Excessive iron can lead to cell death. After cell death, the capsule ruptures, and the contents are released into the tissue, activating the surrounding immune cells that then promote inflammation (Tonnus et al., 2018).

#### Autophagic Cell Death

Autophagy is an evolutionarily and conservatively important process for the turnover of intracellular materials in eukaryotes. Autophagy is divided into macroautophagy (usually called autophagy), microautophagy, and molecular chaperone-mediated autophagy (Jiang and Mizushima, 2013), which generally refers to macroautophagy. The autophagy mentioned in this article refers to macroautophagy. Under normal circumstances, nerve cells continuously maintain a very low level of autophagy (Rubinsztein et al., 2005), but in situations such as starvation, ischemia, hypoxia, protein misfolding, or damage to organelles, autophagy will be activated to maintain cell homeostasis (Yorimitsu and Klionsky, 2005; Yang and Klionsky, 2010).

After TBI, the expression of several autophagy flux indicators increased, such as Beclin1, LC3-II/LC3-I ratio, and Atg12–Atg5 conjugates, suggesting an upregulation of autophagy formation (Diskin et al., 2005; Clark et al., 2008; Liu et al., 2008; Sadasivan et al., 2008). Au et al. (2016) performed LC3 immunohistochemistry on damaged brain tissue from patients, showing that the neurons, oligodendrocytes, and microglia in the damaged area of patients undergoing decompression of the cranial flap after TBI had autophagosome formation, and the increase in P62 was correlated with adverse results 6 months after TBI. This result is consistent with the results of autopsy on TBI patients by Sakai. The findings by Sakai demonstrated that the expression of P62 in neurons and glial cells increased within 1 h after TBI, P62 and LC3 immunohistochemical staining was higher in the contused site than in the non-contused site, and the expression of autophagy markers increased for a long time—or even months after injury (Sakai et al., 2014).

The mechanism of autophagy flux change after TBI is not very clear. One mechanism is brain iron accumulation. Autophagy occurs after cerebral hemorrhage and that using an iron chelator significantly reduces cerebral hemorrhage-induced autophagy. It is suggested that iron plays an important role in inducing autophagic cell death after cerebral hemorrhage, and this effect may be one of the secondary injury mechanisms of the brain (He et al., 2007). Iron overload changes the size and number of lysosomes and affects autophagic flux (Fernández et al., 2016). The increased free iron pool generates ROS through the Fenton reaction (Dowdle et al., 2014). H_2_O_2_ produced by oxidative stress can bind to the cysteine 81 site of ATG4 to regulate the activity of ATG4 (Scherz-Shouval et al., 2007), then enhance autophagy and cause autophagic cell death (Asnaghi et al., 2004; Koike et al., 2008). At the same time, increased autophagy can degrade ferritin by lysosome and increase intracellular iron levels (Dong et al., 2008), forming a negative feedback.

#### Ferroptosis

Ferroptosis is a form of regulated cell death that occurs as a consequence of lethal lipid peroxidation (Stockwell et al., 2017). It is due to the inactivation of the glutathione (GSH)-dependent antioxidant defense mechanism in cells, and the ability of GPX4 to suppress lipid ROS accumulation is inhibited, which eventually leads to lipid peroxidation and cell death (Dixon et al., 2012).

Three factors are closely related to ferroptosis: iron, polyunsaturated fatty acids, and amino acids. Several studies have shown that iron is necessary for ferroptosis, and excessive iron deposition has been reported after TBI (Raz et al., 2011). The free iron pool in the cytoplasm is the most powerful producer of ROS. The brain is the most abundant source of polyunsaturated fatty acids and susceptible to lipoxygenase catalysis and ROS oxidation (Cheng and Li, 2007; Skouta et al., 2014). After TBI, the release of glutamate at the terminals of presynaptic neurons increases. High concentrations of extracellular glutamate can inhibit cystine, which is necessary for the synthesis of glutathione entering the cytoplasm by Sx^c–^; pertinently, insufficient GSH inhibits GPX4 activity and causes ferroptosis. At last, iron-induced oxidative stress exceeds the ability of anti-oxidative stress, and ferroptosis occurs.

To date, almost all genes related to iron-induced cell death are regulated by Nrf2 transcription (Abdalkader et al., 2018). Nrf2 is a central regulator that maintains redox homeostasis and regulates the redox state of cells in a harmful state (Villeneuve et al., 2010); it can be transferred to the nucleus under oxidative stress to activate a variety of antioxidant enzymes, such as GPX (de Vries et al., 2008). In the acute stage of cerebral hemorrhage, the expression of GPX4 is significantly reduced, and the increase in GPX4 can improve iron-dependent neuron death in rats and improve the recovery of cerebral hemorrhage (Zhang et al., 2018).

### Cerebrospinal Fluid-Contacting Neurons

Cerebral spinal fluid comprises the inherent contents of the skull mainly produced by the choroid plexus in the ventricle and subarachnoid space. It surrounds and supports brain tissue and the spinal cord in their entirety and plays a role in supporting and removing metabolites. CSF contains a variety of signals that participate in the regulation of central nervous system activities and exchange substances with blood through a variety of ion channels. Brain metabolites are secreted and infiltrated into CSF to regulate the entire central nervous system and even organs throughout the entire body (Illes, 2017).

Recent research suggests that there is a class of neurons in contact with CSF on the ependyma called CSF-contacting neurons (CSF-cNs), and the location of CSF-cNs and ciliary morphology suggests that they sense physical and chemical signals in the CSF (Orts-Del’Immagine and Wyart, 2017). The hippocampus is closely related to learning, memory, and cognitive function. Studies have found that 5-HT can help hippocampal learning and memory (Oliveira-Silva et al., 2007). Through the horseradish peroxidase tracing method, it has been confirmed that CSF-cNs project nerve fibers to the dCA1 area and transmit 5-HT to the hippocampus; they can also transport 5-HT from adjacent cells or CSF to hippocampal neurons. When a CSF-cN is impaired, 5-HT transmission decreases, and cognitive function declines (Li et al., 2020).

**FIGURE 3:**
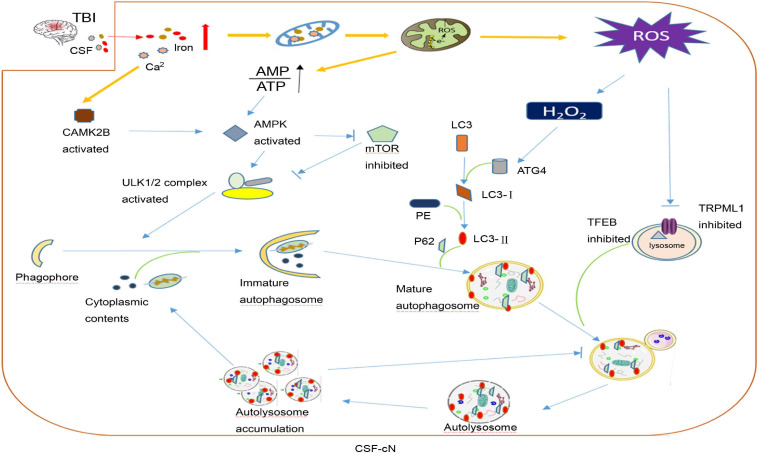
A hypothesis of cerebrospinal fluid-contacting neuron (CSF-cN) autophagy abnormality in traumatic brain injury (TBI). After traumatic brain injury, both iron and calcium are increased in cerebral spinal fluid, which are largely taken by CSF-cNs. Excessive Ca^2+^ activates calcium/calmodulin-dependent protein kinase IIβ (CAMK2B) and increases the permeability of mitochondria resulting in respiratory chain destruction and AMP/ATP ratio increase. Both activated CAMK2B and increased AMP/ATP ratio could activate AMPK to promote the formation of immature autophagosomes by directly activating ULK1 or inhibiting mTOR. Excessive iron in the mitochondria increases the generation of ROS including H_2_O_2._ H_2_O_2_ could combine with cysteine 81 sites of ATG4 to promote lipidation of LC3 to LC3-II, which participates in mature autophagosome formation. In addition, excessive ROS may inhibit lysosomal ion channel TRPML1, which may be one of the mechanisms that mediate the abnormality of CSF-cN autophagy after TBI and transcription factor EB (TFEB) to affect lysosome biogenesis and suppress the hydrolysis ability of the lysosome. At last, autophagosome formation increases and autolysosome hydrolysis is abnormal, both together leading to autophagy flux abnormality and cytoplasmic content accumulation including damaged mitochondria, misfolded protein, etc.

After TBI, CSF-cN iron-induced damage may be part of the reason why cognitive dysfunction develops. In addition to contact with CSF, CSF-cNs also connect with other neurons, glial cells, and blood vessels in the brain parenchyma. As mentioned above, oxidative stress is the main secondary injury in TBI caused by iron overload. Savman et al. (2001) compared CSF non-protein-bound iron in 20 premature infants with ventricular hemorrhage and 10 normal premature infants and found that 75% of intraventricular hemorrhage (IVH) infants had CSF non-protein-bound iron, but none of the infants in the control group had CSF non-protein-bound iron, and all children with CSF non-protein-bound iron suffered white matter damage and subsequent disability. CSF-cNs are highly likely to take a large amount of iron from CSF to generate ROS. Under oxidative stress, CSF-cNs upregulated the activity of ATG4 by combining H_2_O_2_ with the cysteine 81 sites of ATG4 and activated ATG4-promoted LC3 lipidation, which is essential for autophagy initiation, and promoted the formation of autophagosomes (Scherz-Shouval et al., 2007; Lin and Kuang, 2014). TRPML1 (also known as MCOLN1) belongs to the mucoprotein subfamily of transient receptor potential channels and co-express with the CSF-cNs special marker TRPP2 channel in vestibular (Takumida and Anniko, 2010). Recently, the Xu Haoxing team in the United States confirmed that TRPML1 is a Fe^2+^ transport channel in late endosomes and lysosomes. MCOLN1−/− cells show obvious Fe^2+^ deposition in lysosomes and cause cellular iron metabolism disorder and neuronal degeneration (Dong et al., 2008). In TRPML1-deficient mice, an increase in p62 and a clearance defect in LC3-II were found, which shows that the complete autophagy flux was destroyed (Curcio-Morelli et al., 2010). Thus, the abnormal autophagy of CSF-cNs may be part of the pathophysiological mechanism for neurological dysfunction after TBI (Figure 3).

## Clinical Significances for Iron Metabolism Disorders After Traumatic Brain Injury

### Serum Iron and Prognostic Ability

Some studies have shown that serum ferritin is independently associated with severe cerebral edema and poor prognosis after cerebral hemorrhage. Serum iron and transferrin levels were negatively correlated with hematoma volume; serum ferritin levels in the experimental group were significantly higher than those in the control group, and the worse prognosis is when the serum ferritin level is higher. There was no significant difference in serum CP and serum transferrin (Yang et al., 2016). In a 3-year randomized double-blind experiment including 818 elderly individuals, an increase in serum ferritin was significantly correlated with a decline in cognitive function (Schiepers et al., 2010). The research by Gao et al. (2017) also indicated that severe cognitive impairment is associated with elevated serum ferritin. Ferritin is an acute phase protein of the inflammatory response (Marshall, 2001), and the increase in ferritin in inflammation causes a decrease in serum iron (Drakesmith and Prentice, 2012). At the same time, hepcidin is also regulated by inflammation, and the expression of serum hepcidin in patients with cerebral hemorrhage is increased (Ganz and Nemeth, 2012). Mecklenburg confirmed the correlation between serum ferritin and serum hepcidin in the case of inflammation (Mecklenburg et al., 2014). In general, the combined application of serum ferritin, hepcidin, and iron indexes may be more clinically meaningful for patients with hematoma after TBI.

### Intervention Strategies and Clinical Trials

For patients who meet the surgical standards, posttraumatic intracranial hematoma (TICH) removal can reduce the neurotoxic effect of iron released by lysis from red blood cells (Tao et al., 2017) and prevent the occurrence of cerebral hernia caused by the increase in intracranial pressure (Arboix et al., 2002). The hematoma removal technique used for TICH varies around the world. Although there are clinical trials for spontaneous cerebral hemorrhage removal, there are still no clinical trials specifically for TICH hematoma removal (Table 1). As early as the 1990s, Zumkeller et al. (1992) found that the incidence of undesirable prognosis in the hematoma removal group of TICH patients was better than that in the non-surgical group of TICH patients. Similarly, in a single-center retrospective study, Choksey et al. (2009) found that in patients with a low GCS score whose TICH hematoma volume was greater than 16 ml, the proportion of patients with undesirable prognosis in the hematoma removal group was 38% less than that in the non-surgical group (56%). Open surgery may cause damage to offset the benefits of clearing the hematoma; thus, research to determine minimally invasive hematoma removal strategies are currently underway (Wilkinson et al., 2018), such as that evaluated in the MISTIE III experiment, endoscopic hematoma removal, and the stereotactic underwater blood aspiration (sCUBa) (Turner et al., 2015; Kellner et al., 2018).

**TABLE 1:** Intervention strategies and clinical trials for iron overload in traumatic brain injury (TBI).

Strategy		Name	Current use	Preclinical studies	Clinical studies	Deficiency	References
Surgical clot removal	Open surgery/Craniotomy	Hematoma removal	Reduce the neurotoxic effect of iron released by lysis from red blood cells. Prevent the occurrence of cerebral hernia	No preclinical trial specifically for TICH hematoma removal	Undesirable prognosis in the hematoma removal group is better than that of the non-surgical group (low GCS patients with TICH hematoma volume >16 ml)	Open surgery may bring damage to offset the benefits of clearing hematoma	Zumkeller et al., 1992; Choksey, 2009
	Minimally invasive methods	Minimally Invasive Surgery Plus Alteplase for Intracerebral Hemorrhage Evacuation III (MISTIE III)	Primary end point was good functional outcome at 365 days (defined as a modified Rankin Scale score of 0–3). The trial-defined surgical aim was to reduce the hematoma to (=15 ml)		(1) A study Including <15 adults with supratentorial spontaneous ICH had no positive result; (2) MISTIE III demonstrated a benefit in mortality (19% in surgical group vs. 26% in the medical group); (3) 53.1% of the 145 surgical patients with end of treatment (EOT) goal ≤ 15 ml achieved good functional outcome (mRS 0–3) at 1 year, which is more than 32.7% of the 101 patients with >15 ml at EOT	Failed to show superiority to standard medical care. Do not permit clear visualization of residual clot burden, nor do they permit reliable visualization and cauterization of active arterial bleeding. Need further evaluation and comparison to determine its relative safety and efficacy	Awad et al., 2019; Hanley et al., 2019
		Endoscopic hematoma removal	Prevent the damage brought by open surgery. Reduce the neurotoxic effect of iron released by lysis from red blood cells		(1) 43% patients in surgical group with a good neurological outcome at 6 months versus 24% in the medical group (*n* = 20). (2) Multivariate analysis revealed the operative time was significantly decreased in the endoscopy group compared with that of the craniotomy group (*p* < 0.001)	No more trials	Vespa et al., 2016; Katsuki et al., 2020
		Stereotactic underwater blood aspiration (sCUBa)			Clear identification and cauterization of bleeding vessels and visualization of residual clot burden in 47 patients		Kellner et al., 2018
Endogenous clearance of hematoma		Pioglitazone(PIO)	Hematoma resolution		A phase 2 randomized, blinded, placebo-controlled study, to assess the safety of PIO (in increasing doses from 0.1 to 2 mg/kg/day for 3 days followed by a lower maintenance dose)		Gonzales et al., 2013
Iron chelators		Desferrioxamine (DFO)	Acute iron posioning, iron chelation for chronic iron overload	(1) Delayed erythrocyte lysis, reduced iron deposition, oxygen species generation, heme oxygenase-1 expression, and alleviated neuron degeneration and myelin sheath injury in ICH (100 mg/kg). (2) Deferoxamine given 2 h after ICH reduced free iron in CSF at all time points (100 mg/kg, i.p., administered 2 h after ICH and then at 12-h intervals for up to 7 days)	Deferoxamine mesylate may accelerate hematoma absorption and inhibit edema in TICH patients (*n* = 94);	Need more advantage evidence to move DFO forward to Phase III clinical trial	Wan et al., 2006; Yu et al., 2017; Guo et al., 2019
		Deferiprone (L1)	Iron chelation for chronic iron overload	Deferiprone reduced iron contents but not brain water content and ROS generation in ICH rats	NO	Failed to improve the outcome in preclinical trials and no clinical trials related with TBI	Wang et al., 2016
		Deferasirox (ICL670)		*In vitro* studies of cyclodextrin–desferasirox conjugate have shown improvement in anti-oxidant activity and inhibition of metal (iron)-induced protein aggregation with less cytotoxicity			Gascon et al., 2019

Twenty-four hours after bleeding, iron begins to be released into the brain; on the seventh day, the non-heme iron content of the brain triples and remains at a high level for at least 28 days. This is an important time window for the treatment of secondary damage caused by blood catabolites. The first step is to promote the absorption of the endogenous hematoma and reduce the continuous decomposition of red blood cells to prevent the release of toxic substances such as iron. In a clinical trial, the use of the FDA-approved peroxisome proliferator-activated receptor (PPAR)-g agonist pioglitazone can promote the endogenous absorption of hematoma (Gonzales et al., 2013). In addition, in animal model studies, the enhancement of the activity of low-density lipoprotein receptor-related protein-1 (LRP1) and the inhibition of the expression of erythrocyte integrin-related protein CD-47 can enhance endogenous hematoma clearance (Cao et al., 2016). These elements may all become potential therapeutic targets for clinical endogenous hematoma removal. Although a previous study has confirmed that hematoma removal is beneficial for improving cognitive function of cerebral hemorrhage patients (Tyler et al., 1999), not all hematoma removal show outcome benefit. In the clear III trial administrated by the Daniel F. Hanley team, the outcome shows a reduced mortality of IVH patients after alteplase treatment, but does not improve the secondary neurological dysfunction significantly (Hanley et al., 2017). In view of the uncertain benefit of hematoma removal, researchers and neurosurgeons then put the treatment targets on overload iron and the secondary injury induced by iron. So, the second step is the use of an iron chelator. For this step, deferoxamine has been used for more than 40 years. It mainly forms stable compounds with ferric iron and hemosiderin to reduce the oxidative stress caused by iron to play a neuroprotective role (Zaman et al., 1999). It has the characteristics of fewer side effects and can quickly concentrate in the brain tissue through the BBB (Palmer et al., 1994). An injection dose of 62 mg/kg/day for three consecutive days to treat patients with cerebral hemorrhage proved to be safe and tolerated (Selim et al., 2011). Magdy found that the use of deferoxamine can reduce serum oxidative stress markers and increase antioxidant capacity (Selim, 2008). In a controlled experiment by Yu et al. (2017) on 94 posttraumatic cerebral hemorrhage patients, the size of edema on days 3, 7, and 14 in the deferoxamine group was significantly smaller than that in the unused group, confirming that deferoxamine can promote the absorption of hematoma in the short term. Currently, deferoxamine is undergoing a second phase clinical trial (Yeatts et al., 2013). There are three iron chelators, desferrioxamine, deferiprone, and desferasirox, for the treatment of β-thalassemia (Ward et al., 2015). At present, there are also a large number of experiments to study its application in neurodegenerative diseases. Due to oxidative stress caused by iron and ferroptosis caused by excitatory amino acid synergy, a large number of clinical trials on anti-amino acid toxicity and antioxidative stress treatment have been conducted. Importantly, selfotel is a glutamate antagonist. In the second phase of human experiments, we found that the mortality caused by its protective treatment was higher than that of the control group (Davis et al., 2000). Additionally, cerestat is a non-competitive glutamate antagonist with good drug tolerance in human experiments because the number of patients in most experimental centers is too small, greatly reducing the validity of the experimental data (Narayan et al., 2002). Some endogenous and synthetic antioxidants, such as polyethylene glycol absorbed (PEGSOD), tirilizad (Upjohn), and vitamin E, have been studied for antioxidant effects, but none have shown therapeutic effects (Hawryluk and Bullock, 2015).

In consideration of the relevance between CSF-cNs and cognitive function (mentioned above), although there is no clinical trial, some rodent animal model and cellular-level studies give us a preliminary understanding. The Li Qing team used the cholera toxin subunit (CB)-saporin (SAP) into the lateral ventricle of rats to exclusively damage the CSF-cNs. As a result, learning and memory abilities in animals decreased significantly after the destruction of CSF-cNs (Li et al., 2020). We have demonstrated that the brain is with acidosis after TBI in the *Current Understanding of Secondary Brain Injury After Traumatic Brain Injury* section. Actually, the unpublished data of our team indicates that acidosis can induce neural stem cell damage through excessive activated acid-sensing ion channels (ASICS), which are abundantly located on the dendritic spine of CSF-cNs. As a specific ASIC1a blocker, Psalmotoxin 1 (PcTX1) injected in the ventricular could reduce the focal infarct size up to 60% forever or temporarily in the cerebral ischemia model (Xiong et al., 2004). In general, CSF-cNs could be a new treatment target after TBI.

## Perspective

It is very clear that iron overload and iron metabolism-related protein disorder is closely related to mTBI. Iron overload can produce autophagic death and ferroptosis, and promote the phosphorylation of tau protein to form nerve fiber tangles, increasing the risk of TBI patients suffering from neurodegenerative diseases such as Alzheimer’s disease and Parkinson’s disease; In addition, it also can induce neural network damage.

However, there is little research about iron accumulation in the TBI injury mechanism. A single research strategy resulted in few effective treatments for nerve damage caused by iron toxicity. A large number of studies found that cell damage caused by iron overload cannot be alleviated by antioxidant or anti-excitatory amino acid treatment, and it was not very helpful for the improvement of cognitive function and the long-term recovery of the patient. Many related agents have played a role in preclinical animal experiments but rarely can be converted into clinical applications. In the early stage, some effects of iron chelators, such as deferoxamine, have been found in anti-iron therapy, but they have not yet been formally investigated for the clinical treatment of TBI patients. In mechanistic research, various research centers around the world have adopted a variety of experimental animal models to simulate the pathophysiological characteristics of human TBI. A large number of neuroprotective agents have been found in animal and cell brain injury models, and none of them have shown positive long-term results in clinical trials (Menon, 2009; Agoston et al., 2012). Most preclinical studies focus on the acute and short-term pathological effects of TBI (Agoston et al., 2012), but ignoring mTBI, which is the large proportion of TBI, and mTBI’s clinical characteristics are always with long-term emotional and cognitive dysfunction. The clinical characteristics of TBI are extremely heterogeneous, and all animal models possessed only a few pathological features (DeWitt et al., 2018). For example, the chronic traumatic encephalopathy experimental animal model cannot replicate the typical pathological features of human chronic traumatic encephalopathy: phosphorylation of tau protein and the formation of nerve fiber tangles. Most of the current multicenter preclinical trials on TBI are not effective. In future research, we should seek better methods for making animal models that are characterized by pathophysiological mechanisms closer to human TBI patients and should be replicated in large experimental animals.

## Conclusion

Iron overload has an extremely important role in the secondary injury of mTBI. Autophagy exists in various nerve cells, and the effect of autophagy inhibition or activation on the nervous system after brain trauma is not yet clear, but it has been confirmed that the expression of autophagy formation-related proteins increases. We assume that CSF-cNs suffer from iron accumulation, and the autophagy flux changed, and maybe an important mechanism of cognitive dysfunction after mTBI. In future research, we should further explore the development of autophagy and the interaction mechanism with iron after mTBI and seek new targets for intervention. The CSF-cN neurotransmitter transmission can affect the learning and memory ability of the hippocampus. Our research team has confirmed that iron deposition around the ventricle and ependymal cilia damage occur after IVH, potentially representing a new TBI treatment target, and may be related to the long-term recovery of cognitive function.

## Author Contributions

SH, SL, and YC drafted the manuscript and figures. HF and YC contributed to proofread and revised the manuscript. YC gave the final proof for this submission. All the authors contributed to the article and approved the submitted version.

## Conflict of Interest

The authors declare that the research was conducted in the absence of any commercial or financial relationships that could be construed as a potential conflict of interest.
